# Prophages are infrequently associated with antibiotic resistance in *Pseudomonas aeruginosa* clinical isolates

**DOI:** 10.1128/msphere.00904-24

**Published:** 2025-02-13

**Authors:** Tony H. Chang, Julie D. Pourtois, Naomi L. Haddock, Daisuke Furukawa, Kate E. Kelly, Derek F. Amanatullah, Elizabeth Burgener, Carlos Milla, Niaz Banaei, Paul L. Bollyky

**Affiliations:** 1 Division of Infectious Diseases and Geographic Medicine, Department of Medicine, Stanford University School of Medicine, Stanford, California, USA; 2 University of California San Francisco Medical School, San Francisco, California, USA; 3 Department of Orthopaedic Surgery, Stanford Hospital and Clinics, Redwood City, California, USA; 4 Division of Pediatric Pulmonology and Sleep Medicine, Children’s Hospital Los Angeles, Keck School of Medicine at USC, Los Angeles, California, USA; 5 Cystic Fibrosis Clinic, Department of Medicine, Lucille Packard Children’s Hospital, Stanford University School of Medicine, Stanford, California, USA; University of Nebraska Medical Center College of Medicine, Omaha, Nebraska, USA

**Keywords:** antimicrobial activity, bacteriophage genetics, bacteriophage therapy, antimicrobial safety

## Abstract

Lysogenic bacteriophages can integrate their genome into the bacterial chromosome in the form of a prophage and can promote genetic transfer between bacterial strains *in vitro*. However, the contribution of lysogenic bacteriophages to the incidence of antimicrobial resistance (AMR) in clinical settings is poorly understood. Here, in a set of 186 clinical isolates of *Pseudomonas aeruginosa* collected from respiratory cultures from 82 patients with cystic fibrosis, we evaluate the links between prophage counts and both genomic and phenotypic resistance to six anti-pseudomonal antibiotics: tobramycin, colistin, ciprofloxacin, meropenem, aztreonam, and piperacillin–tazobactam. We identified 239 different prophages in total. We find that *P. aeruginosa* isolates contain on average 3.06 ± 1.84 (SD) predicted prophages. We find no significant association between the number of prophages per isolate and the minimum inhibitory concentration for any of these antibiotics. We then investigate the relationship between particular prophages and AMR. We identify a single lysogenic phage associated with phenotypic resistance to the antibiotic tobramycin and, consistent with this association, we observe that AMR genes associated with resistance to tobramycin are more likely to be found when this prophage is present. However, we find that they are not encoded directly on prophage sequences. Additionally, we identify a single prophage statistically associated with ciprofloxacin resistance but do not identify any genes associated with ciprofloxacin phenotypic resistance. These findings suggest that prophages are only infrequently associated with the AMR genes in clinical isolates of *P. aeruginosa*.

**IMPORTANCE:**

Antibiotic-resistant infections of *Pseudomonas aeruginosa* (*Pa*), a leading pathogen in patients with cystic fibrosis (CF), are a global health threat. While lysogenic bacteriophages are known to facilitate horizontal gene transfer, their role in promoting antibiotic resistance in clinical settings remains poorly understood. In our analysis of 186 clinical isolates of *P. aeruginosa* from CF patients, we find that prophage abundance does not predict phenotypic resistance to key antibiotics but that specific prophages are infrequently associated with tobramycin resistance genes. In addition, we do not find antimicrobial resistance (AMR) genes encoded directly on prophages. These results highlight that while phages can be associated with AMR, phage-mediated AMR transfer may be rare in clinical isolates and difficult to identify. This work is important for future efforts on mitigating AMR in CFCF and other vulnerable populations affected by *Pa* infections and advances our understanding of bacterial-phage dynamics in clinical infections.

## OBSERVATION

Mobile genetic elements such as plasmids, transposable elements, and integrons play an important role in the dissemination of AMR genes across bacterial populations via horizontal gene transfer. Plasmids, in particular, often carry multiple resistance genes that confer multidrug resistance to their bacterial hosts (1, 2). Transposable elements, including insertion sequences and transposons, likewise facilitate the movement of resistance genes and contribute to the spread of AMR (3–5).

Bacteriophages (phages), viruses that infect bacteria, represent another form of mobile genetic element occasionally implicated in AMR transfer. Bacteriophages can contribute to the transmission of AMR genes through transduction, a process where bacterial DNA is packaged into phage particles and delivered to new host cells (6, 7). However, while phage-mediated transduction has been observed in the lab (6, 8, 9), its relevance to clinical AMR is still unclear.

Prophages are lysogenic bacteriophages that remain integrated within the bacterial chromosome until induced to enter the lytic cycle, at which time new phage particles are produced and the cell is lysed (10, 11). Few phages directly encode AMR genes (6, 12–14); however, phages have been shown to contribute to the spread of AMR through either generalized or specialized transduction (12, 15–17). Generalized transduction is the mispackaging of bacterial DNA into the phage capsid and is considered relatively uncommon (18). Specialized transduction, on the other hand, occurs when the prophage is excised with some of the adjoining bacterial genetic material (19, 20).

Here, we investigate the relationship between the presence of prophages and both genotypic and phenotypic AMR in the setting of clinical isolates of *Pa* collected from patients with CF, a genetic disease often associated with chronic bacterial respiratory infections. We use whole-genome sequences and MIC measurements for 186 clinical isolates of *Pa* from 82 patients seen at the Cystic Fibrosis Center at Lucille Packard Children’s Hospital at Stanford University Medical Center. While prophage count is not a direct proxy for transduction, we hypothesize that a high number of prophages may broadly reflect a higher susceptibility to phages, for example, through the conservation of common phage receptors, and, thus, a higher rate of transduction and AMR transmission. Finally, we test directly whether prophages encode for AMR genes.

We first identified all prophages integrated into the genomes of the *Pa* clinical isolates. Prophages were then assigned the same identification number if they were a similar length (within 10% of each other) and showed at least 90% pairwise similarity. We found that the distribution of prophage abundance varied with most isolates containing between one and four prophages (mean = 3.06 ± 1.84) (Fig. S1). Isolates harboring two prophages were most common, followed closely by those with one prophage. The occurrence of isolates with five or more prophages decreased steeply with only a few instances of isolates containing up to 10 prophages, which was the maximum number we observed. All isolates had at least one prophage.

The majority of prophages appeared only once or a few times, indicating a high diversity of prophages among the isolates, with only a few prophages present in more than 10 isolates (Fig. S1). The most common prophage was found in 53 isolates out of 186 clinical isolates. This suggests that while a few prophages may be common, the prophage population is predominantly composed of diverse, low-frequency variants. We identified 239 different prophages in total.

We measured the MIC of clinical isolates and categorized them into susceptible, intermediate, and resistant isolates for tobramycin, colistin, meropenem, ciprofloxacin, aztreonam, and tazobactam/piperacillin. We found that 38%, 11%, 42%, 56%, 25%, and 19% of isolates were either intermediate or resistant to tobramycin, colistin, meropenem, ciprofloxacin, aztreonam, and tazobactam/piperacillin, respectively.

### The number of prophages is not associated with phenotypic resistance to six commonly used antibiotics

**Fig 1:**
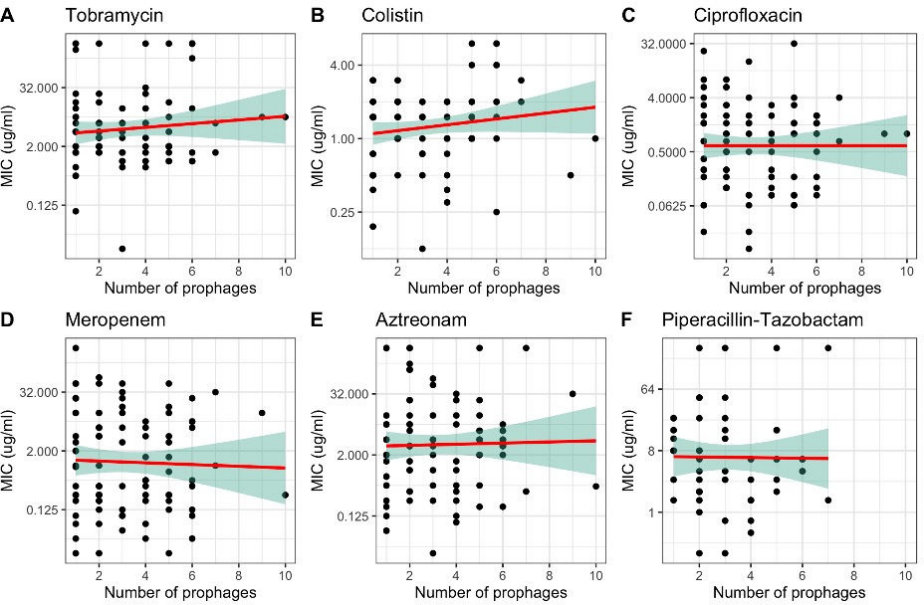
There is no relationship between the number of prophages and the minimum inhibitory concentration (MIC) for various antibiotics. (A) Tobramycin, (B) colistin, (C) ciprofloxacin, (D) meropenem, (E) aztreonam, and (F) piperacillin–tazobactam. All the *y*-axes are on a log2 scale. Each plot includes the linear regression line (red) with a shaded 95% confidence interval (green). None of the slope coefficients are significantly different from 0 (*P* > 0.05).

We hypothesized that a high number of prophages in an isolate may be used as a proxy for higher levels of transduction and be associated with high phenotypic AMR. We tested this association for six antibiotics commonly used to treat *Pa* infections in patients with CF: tobramycin, colistin, ciprofloxacin, meropenem, aztreonam, and piperacillin–tazobactam. There was no significant association between the number of prophages per isolate and the MIC for any of these antibiotics (Fig. 1, *t*-test, *P* > 0.05). This suggests that prophage abundance alone is not a major factor influencing resistance profiles in these bacterial isolates.

### Specific prophages are associated with an increase in phenotypic resistance to tobramycin and ciprofloxacin

Most prophages were not present in enough isolates to evaluate their relationship with resistance. We selected the four most prevalent phages (*N* > 20), which we named vB_Tem_CfSt1-4, and evaluated their association with the MIC for the four antibiotics tobramycin, colistin, ciprofloxacin, and meropenem. We found no association between these prophages and resistance to colistin or meropenem (Fig. S2).

**Fig 2:**
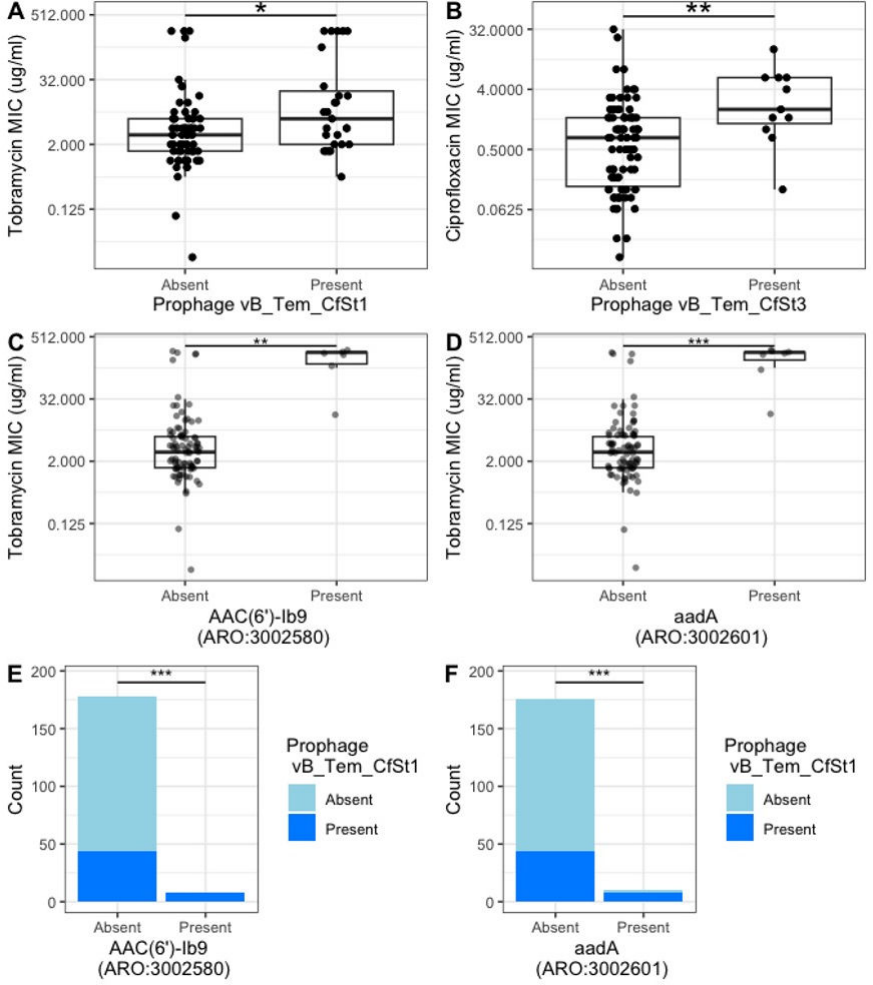
Identification of a pair of prophages that are associated with AMR. We selected the four most prevalent phages which we named vB_Tem_CfSt1-4 and evaluated their association with the MIC of four antibiotics tobramycin, colistin, ciprofloxacin, and meropenem. We found no association between these prophages and resistance to colistin or meropenem (Fig. S2). However, we observed a significant increase in tobramycin MIC when prophage vB_Tem_CfSt1 (A) was present and a significant increase in ciprofloxacin resistance when prophage vB_Tem_CfSt3 was present (B). *, **, and *** for *P*-values smaller than 0.05, 0.01, and 0.001, respectively. (C) Tobramycin resistance (tobramycin MIC on a log2 scale) for isolates with and without resistance genes AAC(6')-Ib9 (ARO:3002580). (D) Tobramycin resistance (log2 tobramycin MIC) for isolates with and without resistance genes aadA (ARO:3002601). (E) Presence of temperate phage vB_Tem_CfSt1 for isolates with and without antibiotic resistance genes AAC(6')-Ib9. (F) Presence of temperate phage vB_Tem_CfSt1 for isolates with and without antibiotic resistance genes aadA. We use *, **, and *** for *P*-values smaller than 0.05, 0.01, and 0.001, respectively.

However, we observed a significant increase in tobramycin resistance when prophage vB_Tem_CfSt1 (Fig. 2A) was present and a significant increase in ciprofloxacin resistance when prophage vB_Tem_CfSt3 was present (Fig. 2B).

### Prophage associated with increases in tobramycin MIC is also associated with AMR genes

We next asked if the same prophages were associated with the presence of AMR genes. Among all AMR genes described in the CARD-RGI data set found in our isolates, only the genes aadA and AAC(6′)-lb9 were significantly associated with higher MIC to tobramycin (Fig. 2C and D; Wilcoxon test, *P* < 0.01). Finally, we found that the presence of prophage vB_Tem_CfSt1 was significantly associated with the presence of these genes (Fig. 2E and F; Fisher’s test, *P* < 0.01).

The genes aadA and AAC(6′)-lb9 both encode aminoglycosides-modifying enzymes (21, 22). These enzymes are some of the most widespread mechanisms for tobramycin resistance and are known to be highly mobile (23). Other common mechanisms for tobramycin resistance include other mobile genes such as efflux pumps and ribosome methyltransferases. Mutations in the ribosome genes targeted by aminoglycosides are rarer on the other hand.

This is in contrast to ciprofloxacin resistance, which is often acquired through mutations of the A subunit DNA gyrase targeted by quinolones like ciprofloxacin, rather than mobile genetic elements (24). We did not find any genes associated with a higher MIC for ciprofloxacin. Other works also show a lower association between known genotypic markers of resistance and phenotypic resistance for ciprofloxacin compared to other antibiotics like aminoglycosides (25), which is consistent with our findings. Phage-mediated transduction of AMR genes may, thus, be more relevant to antibiotics for which mobile genes, rather than point mutations, confer resistance and will be easier to detect for antibiotics with strong genotype-to-phenotype associations for antibiotic resistance.

### Prophages do not directly encode AMR genes

Finally, we looked for AMR genes in the prophage sequences using the homolog model of the CARD-RGI database. None were identified except for a single occurrence of rsmA, a virulence gene that can also confer antibiotic resistance (26). This is consistent with previous work showing that AMR genes are rarely encoded directly by phages (12).

We were not able to determine the relative location of prophages and antibiotic resistance genes due to the small size of many scaffolds containing prophages and AMR genes. The genome reconstructions from the short-read sequencing performed for this work did not allow us to establish the relative locations of prophages and AMR genes. It is, thus, important to note that the association between prophage vB_Tem_CfSt1 and genes aadA and AAC(6′)-lb9 does not imply causation and could arise from phylogenetic correlation for example. In addition, our findings are limited by low prevalence for many prophages and AMR genes, the majority of which were only found in one isolate. Very few phages were found in more than 20 isolates, and many AMR genes associated with tobramycin resistance were either present in all or very few isolates. The low prevalence of most AMR genes also prevented us from assessing additive effects from the presence of multiple genes.

In summary, we identified a single prophage associated with higher MIC for tobramycin and found that known AMR genes conferring resistance to tobramycin were more likely to be found in isolates infected with that phage. Prophage abundance was not associated with antibiotic resistance. These findings suggest that the spread of AMR through prophages is rare overall. Future work should focus on new phenotype prediction tools to increase sample sizes, as well as long-read sequencing to explore causal relationships between phage and AMR through the relative locations of mobile elements in bacterial genomes.

### Sample collection

From June 2020 to June 2023, 186 *Pa* isolates from respiratory cultures of CF patients were collected at Stanford Hospital under IRB approval #11197. Samples were biobanked with patient consent and de-identified using unique codes.

### DNA extraction and sequencing

DNA was extracted from *Pa* isolates using the DNeasy Blood and Tissue Kit (Qiagen, 69504) and sequenced on an Illumina NovaSeq (100 bp paired-end) and an Illumina NextSeq (150 bp paired-end). The extraction involved bacterial lysis, DNA purification, and elution, ensuring high-quality DNA suitable for sequencing. Sequencing reads were quality-checked using FASTQualityControl and trimmed with Trimmomatic 0.39 or “trim galore” to remove Nextera adapters from raw reads sequenced on Illumina NextSeq. Quality reports were assembled using MultiQC55. Trimmed reads were then assembled with SPAdes using –isolate and --cov-cutoff auto (27).

### Prophage identification

We identified prophages using VIBRANT (28). We then performed a BLAST search (word size = 28 and e-value = 0.005) on all predicted prophages against their own sequences. Prophages were grouped under the same name if they were of similar length (less than 10% difference in length) and showed at least 90% pairwise identity.

### Antibiotic susceptibility testing

Clinical isolates were streaked on LB agar plates and incubated at 37°C overnight. The next day, the colonies were picked and suspended in 2 mL of sterile 0.85% saline solution. The inoculum was vortexed and its turbidity was adjusted until it reached 0.5 McFarland standard (OD 0.08–0.1). The standardized bacterial suspension was evenly streaked onto 150 mm Mueller Hinton agar plates (Thermo Fisher Scientific) using a sterile cotton swab. The plates were allowed to dry for 15 min, and the E test strips (Fisher Scientific) were placed onto the plates and incubated at 37°C. The MICs were read after 16–20 h. MICs were obtained for 93 clinical isolates.

### Identification of AMR genes

Antibiotic resistance genes were identified with a BLAST search (word size = 28, culling_limit = 1, and e-value = 0.005) of bacterial genomes against the homolog model of the CARD-RGI database. This model includes sequences that are determinants of resistance without mutation.

### Statistical analysis

Statistical analyses were performed using R. Tests used include *t*-tests for linear regression, Wilcoxon test, and Fisher’s test. Sample sizes were not predetermined. Investigators were blinded during DNA extraction, library preparation, and phage genome annotation, but not during the final analysis.

## SUPPLEMENTAL MATERIAL

Supplemental Figures msphere.00904-24-s0001.docx Figures S1 and S2.

ASM does not own the copyrights to Supplemental Material that may be linked to, or accessed through, an article. The authors have granted ASM a non-exclusive, world-wide license to publish the Supplemental Material files. Please contact the corresponding author directly for reuse.

## References

[1] Carattoli A. 2013. Plasmids and the spread of resistance. Int J Med Microbiol 303:298–304. doi:10.1016/j.ijmm.2013.02.001 23499304

[2] Partridge SR, Kwong SM, Firth N, Jensen SO. 2018. Mobile genetic elements associated with antimicrobial resistance. Clin Microbiol Rev 31:e00088-17. doi:10.1128/CMR.00088-17 30068738 PMC6148190

[3] Partridge SR. 2011. Analysis of antibiotic resistance regions in gram-negative bacteria. FEMS Microbiol Rev 35:820–855. doi:10.1111/j.1574-6976.2011.00277.x 21564142

[4] Siguier P, Gourbeyre E, Chandler M. 2014. Bacterial insertion sequences: their genomic impact and diversity. FEMS Microbiol Rev 38:865–891. doi:10.1111/1574-6976.12067 24499397 PMC7190074

[5] Morales G, Abelson B, Reasoner S, Miller J, Earl AM, Hadjifrangiskou M, Schmitz J. 2023. The role of mobile genetic elements in virulence factor carriage from symptomatic and asymptomatic cases of Escherichia coli bacteriuria. Microbiol Spectr 11:e04710-22. doi:10.1128/spectrum.04710-22 37195213 PMC10269530

[6] Colavecchio A, Cadieux B, Lo A, Goodridge LD. 2017. Bacteriophages contribute to the spread of antibiotic resistance genes among foodborne pathogens of the Enterobacteriaceae family - a review. Front Microbiol 8:1108. doi:10.3389/fmicb.2017.01108 28676794 PMC5476706

[7] Koonin EV, Makarova KS. 2013. CRISPR-Cas: evolution of an RNA-based adaptive immunity system in prokaryotes. RNA Biol 10:679–686. doi:10.4161/rna.24022 23439366 PMC3737325

[8] Zinder ND, Lederberg J. 1952. Genetic exchange in Salmonella. J Bacteriol 64:679–699. doi:10.1128/jb.64.5.679-699.1952 12999698 PMC169409

[9] Morse ML, Lederberg EM, Lederberg J. 1956. Transduction in Escherichia coli K-12. Genetics 41:142–156. doi:10.1093/genetics/41.1.142 17247607 PMC1209761

[10] Frost LS, Leplae R, Summers AO, Toussaint A. 2005. Mobile genetic elements: the agents of open source evolution. Nat Rev Microbiol 3:722–732. doi:10.1038/nrmicro1235 16138100

[11] Touchon M, Bobay L-M, Rocha EPC. 2014. The chromosomal accommodation and domestication of mobile genetic elements. Curr Opin Microbiol 22:22–29. doi:10.1016/j.mib.2014.09.010 25305534

[12] Enault F, Briet A, Bouteille L, Roux S, Sullivan MB, Petit M-A. 2017. Phages rarely encode antibiotic resistance genes: a cautionary tale for virome analyses. ISME J 11:237–247. doi:10.1038/ismej.2016.90 27326545 PMC5315482

[13] Pfeifer E, Bonnin RA, Rocha EPC. 2022. Phage-plasmids spread antibiotic resistance genes through infection and lysogenic conversion. mBio 13:e01851-22. doi:10.1128/mbio.01851-22 36154183 PMC9600943

[14] Calero-Cáceres W, Ye M, Balcázar JL. 2019. Bacteriophages as environmental reservoirs of antibiotic resistance. Trends Microbiol 27:570–577. doi:10.1016/j.tim.2019.02.008 30905524

[15] Davies J, Davies D. 2010. Origins and evolution of antibiotic resistance. Microbiol Mol Biol Rev 74:417–433. doi:10.1128/MMBR.00016-10 20805405 PMC2937522

[16] Muniesa M, Colomer-Lluch M, Jofre J. 2013. Potential impact of environmental bacteriophages in spreading antibiotic resistance genes. Future Microbiol 8:739–751. doi:10.2217/fmb.13.32 23701331

[17] Haaber J, Leisner JJ, Cohn MT, Catalan-Moreno A, Nielsen JB, Westh H, Penadés JR, Ingmer H. 2016. Bacterial viruses enable their host to acquire antibiotic resistance genes from neighbouring cells. Nat Commun 7:13333. doi:10.1038/ncomms13333 27819286 PMC5103068

[18] Volkova VV, Lu Z, Besser T, Gröhn YT. 2014. Modeling the infection dynamics of bacteriophages in enteric Escherichia coli: estimating the contribution of transduction to antimicrobial gene spread. Appl Environ Microbiol 80:4350–4362. doi:10.1128/AEM.00446-14 24814786 PMC4068684

[19] Griffith AJF, Gelbart WM, Lewontin RC, Miller JH. 2002. Modern genetic analysis: integrating genes and genomes. 2nd ed. W. H. Freeman, New York, NY.

[20] Chiang YN, Penadés J, Chen J. 2019. Genetic transduction by phages and chromosomal islands: the new and noncanonical. PLoS Pathog 15:e1007878. doi:10.1371/journal.ppat.1007878 31393945 PMC6687093

[21] Hollingshead S, Vapnek D. 1985. Nucleotide sequence analysis of a gene encoding a streptomycin/spectinomycin adenyltransferase. Plasmid 13:17–30. doi:10.1016/0147-619x(85)90052-6 2986186

[22] Dornbusch K, Miller GH, Hare RS, Shaw KJ. 1990. Resistance to aminoglycoside antibiotics in Gram-negative bacilli and staphylococci isolated from blood. Report from a European collaborative study. J Antimicrob Chemother 26:131–144. doi:10.1093/jac/26.1.131 2211434

[23] Garneau-Tsodikova S, Labby KJ. 2016. Mechanisms of resistance to aminoglycoside antibiotics: overview and perspectives. Medchemcomm 7:11–27. doi:10.1039/C5MD00344J 26877861 PMC4752126

[24] Aldred KJ, Kerns RJ, Osheroff N. 2014. Mechanism of quinolone action and resistance. Biochemistry 53:1565–1574. doi:10.1021/bi5000564 24576155 PMC3985860

[25] Vanstokstraeten R, Piérard D, Crombé F, De Geyter D, Wybo I, Muyldermans A, Seyler L, Caljon B, Janssen T, Demuyser T. 2023. Genotypic resistance determined by whole genome sequencing versus phenotypic resistance in 234 Escherichia coli isolates. Sci Rep 13:449. doi:10.1038/s41598-023-27723-z 36624272 PMC9829913

[26] Mulcahy H, O’Callaghan J, O’Grady EP, Adams C, O’Gara F. 2006. The posttranscriptional regulator RsmA plays a role in the interaction between Pseudomonas aeruginosa and human airway epithelial cells by positively regulating the type III secretion system. Infect Immun 74:3012–3015. doi:10.1128/IAI.74.5.3012-3015.2006 16622241 PMC1459696

[27] Prjibelski A, Antipov D, Meleshko D, Lapidus A, Korobeynikov A. 2020. Using SPAdes De Novo Assembler. Curr Protoc Bioinformatics 70:e102. doi:10.1002/cpbi.102 32559359

[28] Kieft K, Zhou Z, Anantharaman K. 2020. VIBRANT: automated recovery, annotation and curation of microbial viruses, and evaluation of viral community function from genomic sequences. Microbiome 8:90. doi:10.1186/s40168-020-00867-0 32522236 PMC7288430

