# Supplementary material for: Prophages are infrequently associated with antibiotic resistance in Pseudomonas aeruginosa clinical isolates
Source: mSphere. 2025 Feb 13;10(3):e00904-24. doi: 10.1128/msphere.00904-24 (PMC11934324; doi:10.1128/msphere.00904-24)
Supplement: Supplemental Figures — Figures S1 and S2. [file msphere.00904-24-s0001.docx]

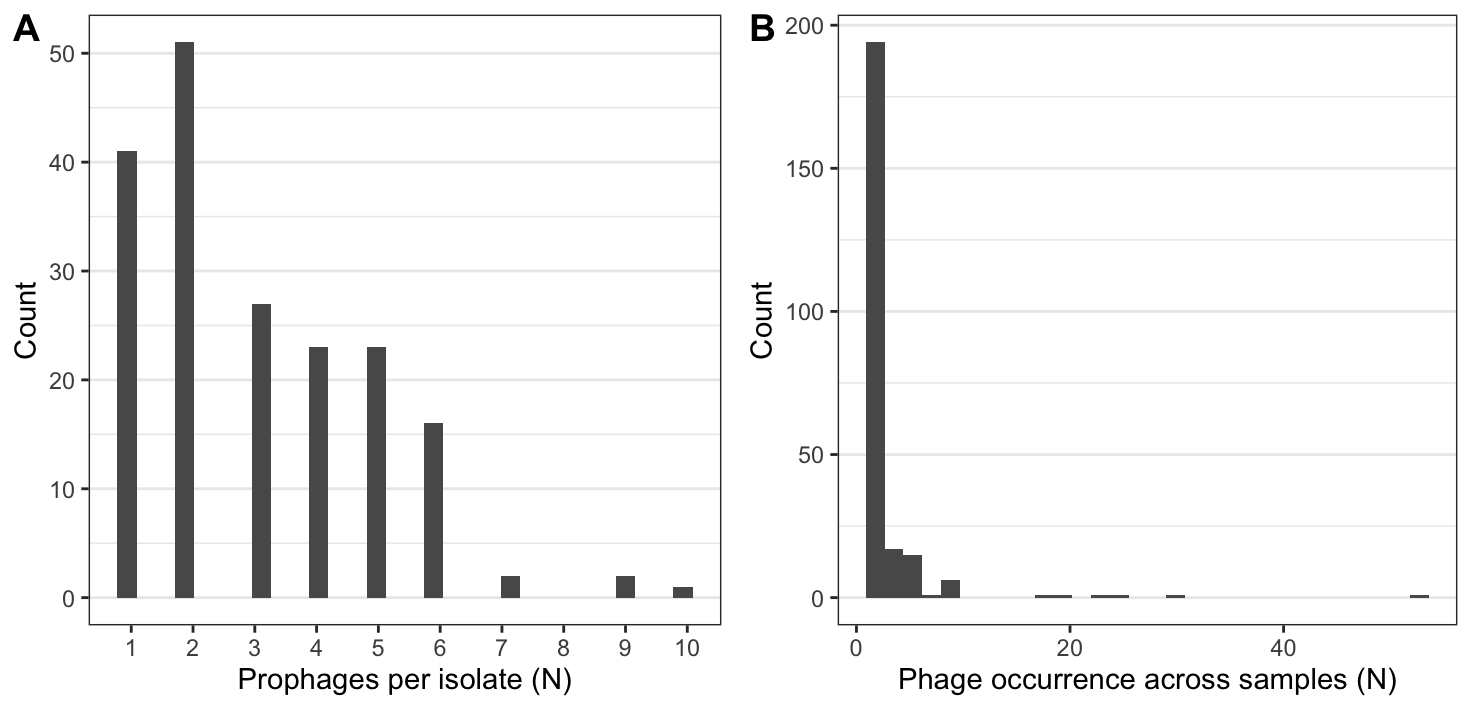


**Figure S1: Prophages are common in clinical samples. A.** Number of prophages per isolate. **B.** Distribution of phage occurrence across the samples. Most phages are present only once or twice with a few phages present many times.


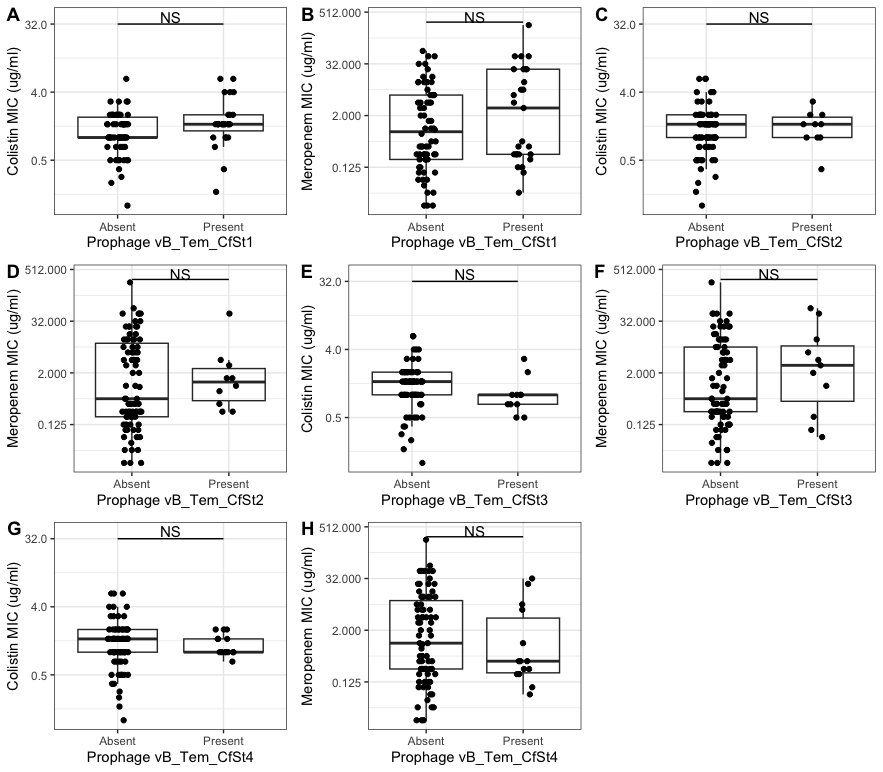


**Figure S2: There was no significant relationship between the presence of any of the 4 most common prophages and phenotypic resistance to colistin (A, C, E, G) or meropenem (B, D, F, H)**.
